# Disasters in pediatric dentistry: a systematic review

**DOI:** 10.1186/s12903-023-03699-0

**Published:** 2023-12-08

**Authors:** Milad Ahmadi Marzaleh, Mohsen Sharif Zadeh Ardakani

**Affiliations:** 1 Department of Health in Disasters and Emergencies, School of Health Management and Information Sciences, Health Human Resources Research Center, Shiraz University of Medical Sciences, Shiraz, Iran; 2 Department of Dental Public Health, School of Dentistry, Shiraz University of Medical Sciences, Shiraz, Iran

**Keywords:** Dentistry, Disasters, Children, Health, Oral, Dental

## Abstract

**Background:**

Disasters can harm many people, especially children, in unpredictable and public ways. One of the neglected aspects of children's health in disasters is oral and dental hygiene, which can affect their physical and mental well-being. This systematic review explores how dentistry can help children in disasters, focusing on two aspects: providing oral health care and identifying disaster victims.

**Methods:**

A thorough search of databases, such as PubMed, Cochrane Library, Scopus, Embase, ProQuest, and Web of Science, was done to find English-language publications from 1930 to August 31, 2023. The screening, data collection, and quality assessment followed the PRISMA guidelines.

**Results:**

Out of 37,795 articles found in the databases, seven research articles were chosen. Five articles were retrospective, and two articles were prospective. The results showed that dentistry for children is very important in disasters by giving information about the oral and dental problems and identifying the victims. The results also showed some of the challenges and difficulties in giving dental care for children in disaster situations, such as changing control, referral systems, and parental fear of infection.

**Conclusion:**

Dentistry for children can improve the health and well-being of children affected by disasters.

## Introduction

Natural disasters, such as floods, cyclones, droughts, heat waves, severe storms, and earthquakes, can harm many people, especially children [1]. Every year, 175 million children around the world are expected to be affected by natural disasters [2]. Natural disasters can affect children's physical health, mental health, and learning in serious ways [3, 4]. One of the aspects of children's physical health that can be harmed by natural disasters is oral and dental health [5]. Dentofacial injuries are injuries that affect the teeth, jaws, or facial bones. They can lead to pain, infection, deformity, or loss of function. Dentofacial injuries can happen because of exposure to physical violence, debris, or explosions during natural disasters [6]. They can also happen because of not having proper oral hygiene and preventive dental care, being malnourished or dehydrated, or being psychologically stressed or traumatized. The prevalence and incidence of dentofacial injuries in children in disaster situations are not well known, especially in low- and middle-income countries [7]. However, some studies have reported the epidemiology, patterns, and mechanisms of pediatric trauma in general, including dentofacial injuries. For example, a retrospective review of 12,508 pediatric trauma patients treated in the emergency department in Germany between 2015 and 2019 found that 2703 fractures, 2924 lacerations and superficial tissue injury, 5151 bruises, 320 joint dislocations, 1284 distortions, 76 burns, and 50 other injuries were treated [8–11]. Therefore, different specialties and disciplines are involved based on valid evidence before, during, and after disasters. Oral and dental hygiene is one of the fields that are often neglected in times of crisis, but it can have severe effects on children's physical and mental health. The role of pediatric disaster dentistry is to prevent, manage, and improve oral and dental problems that may arise from disasters, such as trauma, infection, pain, or psychological issues. Dentists can play a role in disaster management by providing emergency dental services, participating in victim identification through dental evidence, or supporting children's public health and welfare in disaster situations [1, 12, 13].

Some of the roles of dentistry in disasters are providing emergency dental services**,** Assist in victim identification and Supporting general health or well-being in disaster situations. Dentists can provide essential dental care, take preventive measures, identifying deceased victims using records, radiographs, or DNA samples from teeth [14–17], helping collect or preserve evidence from disaster scenes, such as bite marks or tooth fragments [18–20], contributing to the comprehensive care of disaster victims, educate or empower them about the importance of oral hygiene or care in disaster situations [13, 21–23].

Numerous studies have been conducted on the role of dentistry in disasters for different populations and contexts. Nevertheless, no review article has been conducted specifically on the part of dentistry in pediatric disasters. This is an essential gap in studies, as children may require special attention and care from dentists and may benefit from innovative technologies such as teledentistry to increase their motivation, participation, and recovery in disaster situations [24, 25].

Therefore, the present study aims to investigate how dentists can play a role in disaster management and victim identification for children and how they can contribute to oral health education, prevention, and treatment of children affected by disasters in the form of a systematic review in 2023.

## Methods

### Search strategy

We registered our study protocol on the PROSPERO website with the code CRD42023457790. We followed the PRISMA2 guidelines for a systematic review. Our study question was "What is the role of dentistry in children's disasters?" We searched for English-language journals from 1930 to August 31, 2023. We also searched the Cochrane Library database to make sure there were no similar systematic reviews published. We did not find any similar articles. We searched some databases like PubMed, Cochrane Library, Scopus, Embase, ProQuest, and Web of Science. We used the AND operator to compare words that are different concepts, also used the OR operator between synonyms. MeSH terms used in the PubMed database to search for articles (Table 1). We searched the titles, abstracts, and keywords of the articles. The researcher chose the search terms. Then we got the factors from the articles that we chose for analysis. The researchers independently reviewed the articles and after removing the articles that were not related to the subject of the study in terms of the title, abstract and full text of the article, they discussed the disagreements. After mutual discussion and review of the selected articles, they reached a final agreement on the articles with disagreements to exclude or include them in the study. Next, we made a complete list of references for all publications and then we checked the titles of the articles. We removed the articles that were not related to our study goals. To be careful, we did all the search processes twice. We used ENDNOTE X9 software to manage the resources.

**Table 1:** Pediatric dentistry search strategy in disasters

**PIO**	**#1 AND #2 AND #3**	Strategy
P	Child OR Children OR Kid OR " 1–12 years old " OR " school age " OR Toddler OR "Middle Childhood " OR " Early childhood " OR Preschool OR " Grade-schooler " OR Grade schooler OR " preschool " OR preschool OR " school age "	#1
I	Tsunami OR Cyclone OR Tornado OR Hurricane OR " tropical cyclone " OR typhoon OR disaster OR crisis OR emergencies OR emergency OR incident OR " climate change " OR " extreme event " OR catastrophe OR earthquake OR landslide OR flood OR explosion OR " man-made disaster " OR " Man made disaster " OR " technological disaster " OR airplane OR train OR car OR crash OR accident OR CBRNE OR CBRN OR Chemical OR Nuclear OR radiation OR Biological OR Explosive OR Terrorism OR Storm OR Hazard OR Blizzards OR Avalanche OR " Volcanic eruption " OR Bioterrorism OR " Civil unrest " OR Hazardous OR " Rapid Onset disaster " OR Drought OR Landslide	#2
O	dentistry OR dent OR dental OR " dental hygiene " OR " oral hygiene " OR orofacial OR prosthodontic OR prosthodontist OR prosthetic OR restorative OR periodontal OR periodont OR endodontic OR maxillofacial OR exodontist OR orthodontist OR dentofacial OR odont OR stomatology OR tooth OR teeth OR mouth OR " oral cavity " OR " cavitas oris " OR " dental medicine " OR " oral medicine " OR " operative dentistry " OR maxilla OR mandibular	#3

### Inclusion criteria

We first chose articles that had related in the title, keywords, or abstract. Then we checked the abstracts and then we checked the full text using evaluation techniques. We chose articles from 1930 to August 31, 2023. Authors used quantitative and qualitative criteria to choose the best articles for review. The search terms could be found in all section of documents (titles, abstracts or keywords). The articles should be directly related to our study topic. We also included the reviewed articles in our selection process.

### Exclusion criteria

We removed articles that were not in English and also articles that had parameters that were not related to our study purpose. Other exclusion criteria were under 10 participants, studies that are not published in English or do not have an English abstract, studies that are not peer-reviewed or do not have a clear methodology, studies that do not focus on children (aged 0–18 years) or dentistry, studies that do not measure relevant outcomes, such as oral health status, dental trauma, dental anxiety, dental treatment, or disaster victim identification, studies that are duplicates.

### Screening

First, we checked all the article titles in the database to make sure they were relevant. We chose articles that compatible and related our inclusion criteria to our study purpose. Next, the abstracts of the chosen articles checked. In the next step, we chose the articles that were fully in line with our study purpose and inclusion criteria and we read and evaluated their full texts by the authors. Finally, we chose publications that talked about the role of dentistry in pediatric disasters. We evaluated the articles according to PRISMA guidelines. We also considered citation and publication bias and we carefully evaluated articles with high citations. We repeated all these steps twice.

### Collecting data

We made summary forms and collection using Microsoft Word 2016 software and after reading and carefully checking the articles, we got the necessary data from them. This form had sections for title, responsible author, research objectives, population, sample, country, year, design, tools, methods, results, and conclusion of study. We filled out abstract forms for each of the chosen articles. When finished evaluating all articles by authors, all forms put in a table. If there was disagreement, we were asked other authors to say their opinions.

### Ethical considerations

Ethical considerations included logical and scientific analysis of the articles without any bias because this study does not need an informed consent form.

The Shiraz University of Medical Sciences Research Vice-Chancellor gave ethical approval for the project.

## Results

**Fig. 1:**
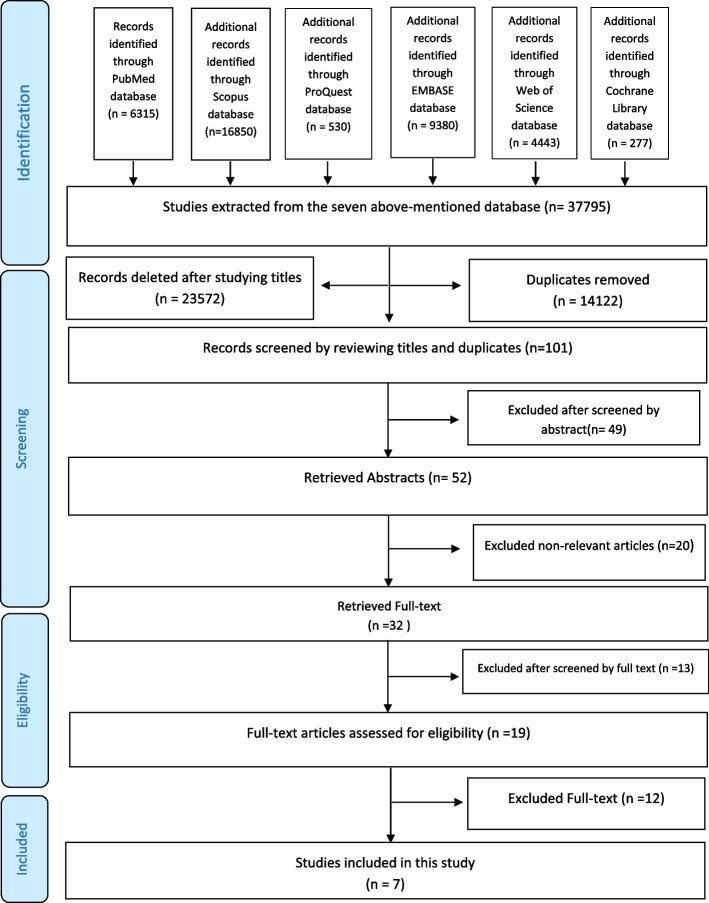
PRISMA flow diagram for the scoping review process

We searched a lot of databases and found 37,795 articles. But we removed 14,122 articles because they were repeated in many databases. We also removed 23,572 articles because their titles had nothing to do with our study goal. After checking all 101 abstracts from before step by step, we removed 49 articles that were not suitable for our research. Finally, we chose 32 full-text articles for our research. Only 7 of these 32 articles met our research goals (Fig. 1). Five of these articles were retrospective (71 0.4%), and two were prospective (28 0.6%) (Table 2). There were also five articles about coronavirus outbreak time. The table shows role of dentistry in pediatric disasters. Summary of the seven articles are in below:

**Table 2:** Type of selected articles

**Study**	**Percentage (%)**	**Number**	**References**
**Retrospective**	71.4	5	[25–29]
**Prospective**	28.6	2	[24, 30]

- The first article compared two methods of estimating the age of individuals based on their dental development and one method based on their skeletal development. It found that the dental methods were similar, but the Cameriere method had a limitation in that it could not estimate dental age beyond 14 years, while the Demirjian method could estimate dental age up to 16 years. It also found that females attained skeletal maturity earlier than males in terms of both chronological and dental age. It suggested that using both the dental and skeletal methods together would be ideal for identifying the age of individuals who are suspected to be between 7 and 16 years old.
- The second article surveyed the opinions and experiences of pedodontists during the COVID-19 pandemic. It found that most of the pedodontists believed that children could transmit COVID-19 without showing any symptoms, and that wearing personal protective equipment (PPE) made it very hard to treat young dental patients. It also found that most of the pedodontists planned to work longer hours, while some of them planned to charge more for their services, to cope with the financial crisis caused by the pandemic.
- The third article evaluated a phone and digital photo service that was used to identify urgent dental problems in children during the COVID-19 lockdown. It found that the service helped to triage the patients and provide them with advice or referrals, but it also faced some challenges, such as not being able to see the patients in person, take X-rays, or use general anesthesia. It suggested that the service could be improved by using video calls, providing more training and support for the staff, and increasing the collaboration with other dental services.
- The fourth article analyzed the data of children and teenagers who received emergency dental treatment in a hospital in Romania between 2019 and 2021. It found that the number of patients decreased from 2019 to 2021, and that the most common age group, tooth type, and type of emergency were 7–12 years old, lower back teeth, and inflammation of the pulp or infection of the root tip, respectively. It also found that younger patients had more injuries to their teeth, and older patients had more problems with their gums. It also found that patients who lived in rural areas had more pulp inflammation or root tip infection, while patients who lived in urban areas had more gum problems.
- The fifth article reviewed the features of the patients, accidents, injuries, and treatments that were similar to what other studies have found in children with dental problems. It found that most of the children who had injuries to their teeth were under 5 years old and boys, and that most of the accidents happened on weekends, at home, and were caused by falls. It also found that more baby teeth and upper front teeth were affected, and that the most common type of injury was when the tooth was pushed sideways. It also found that most of the children were sent to the Children's Dentistry Department, but many of them did not need any further treatment there and were sent back to their regular dentists. It also found that the more serious injuries in the adult teeth tended to be treated between 3 and 12 h after the accident.
- The sixth article reported how professional dentists quickly adjusted to treat children who were not vaccinated during the COVID-19 lockdown. It found that more children came to the emergency service with pain, infection, or ongoing treatment than with injuries during the lockdown, and that these numbers were higher than before and after the lockdown periods. It also found that there was a significant difference in the medication and dental procedures given to the children during the lockdown compared to before and after the lockdown. It also found that pulling out teeth, filling cavities, applying fluoride, and treating the pulp were the main treatments done for the children during the lockdown.
- The seventh article compared the data of children who came to the emergency room (ER) for oral problems in 2019 and 2020. It found that fewer children came to the ER for oral problems in 2020 than in 2019, and that the most common oral problem was inflammation of the mouth, followed by injuries to the teeth, which were less frequent and less common in 2020 than in 2019. It also found that the same percentage of children had to stay in the hospital after visiting the ER in both years, and that there were fewer cases of infectious diseases, except for COVID-19, in 2020 than in 2019, while the number of injuries decreased slightly but the proportion increased. It suggested that there is a need to improve the local services that can prevent and treat oral health problems, including dental emergencies.

The full texts of seven articles are summarized in Table 3.

**Table 3:** A summary form of the ultimately selected papers

N	Correspondence Author/Title	Aim	Population	Sample	Country	Years of Study	Design Study	Type of Disaster	Method	Result	Conclusion
1	J. Kieser/ The usefulness of dental and cervical maturation stages in New Zealand children for Disaster Victim Identification	The study aimed to evaluate two methods of estimating dental age, the Demirjian and Cameriere methods, and to examine the link between dental age and the maturity of the cervical vertebrae (CVM) using the Hassel and Farman method in a sample of New Zealand children	200 children aged between 7 and 17 years	20 participants	New Zealand	2012	Descriptive statistics	Natural and manmade disasters	The methods used in a study that compared different ways of estimating dental and skeletal maturity in 200 children from New Zealand. The researchers used X-rays of the lower jaw and the neck to measure the development of the teeth and the vertebrae. Scored the stages of tooth formation and vertebral shape using three methods: Demirjian, Cameriere, and Hassel and Farman. Checked the accuracy and reliability of their methods by repeating the scoring for 20 random patients after 8 weeks. The statistical tools they used to analyze and compare the data	The Cameriere method had a limitation in that it could not estimate dental age beyond 13.69 years for females and 14.06 years for males, as all seven teeth reached their final stages of development by then. In contrast, the Demirjian method could estimate dental age up to 16 years. Females also attained CVM stages earlier than males in terms of both chronological and dental age. The average chronological age for CVM stages 2–5 was about one year lower in females than in males. The methods of Demirjian and Cameriere for dental maturity and CVM for skeletal maturity were reliable and useful for assessing the age of individuals. For DVI purposes, it would be ideal to use both the dental maturity methods and the CVM method together for estimating the age of individuals who are suspected to be between 7 and 16 years old	The dental methods showed similar results in estimating maturity
2	Nilesh Rojekar/ Knowledge and Perception of COVID-19 among Pedodontists in India: A Quick Online Cross-sectional Study	The study explored the knowledge and perception of COVID-19 among pedodontists in India	pedodontists in India	335 pedodontists	India	2020	A quick online cross-sectional study	COVID-19 PANDEMI	The population and methods used in a study that surveyed 335 pedodontists in India about their knowledge, attitude, and practice regarding the COVID-19 pandemic. The researchers got approval from IEC and used an online platform called "Google Forms" to send a questionnaire to 410 pedodontists in India, out of whom 335 responded. The questionnaire was prepared and validated and had closed-ended questions. The researchers used descriptive statistics and a software called SPSS 22.0 to analyze the data and used a Chi-square test to check the significance of the results. P value of less than 0.05 was considered statistically significant	According to the majority (82%) of the pedodontists who participated in the study, children can transmit COVID-19 without showing any symptoms. More than half (62%) of the pedodontists reported that wearing a personal protective equipment (PPE) kit made it very hard to treat young dental patients. To cope with the financial crisis caused by the pandemic, most (64%) of the pedodontists planned to work longer hours, while some (36%) of them planned to charge more for their services	Preventing COVID-19 transmission is a critical responsibility of pedodontists
3	N. Patel/ Pediatric dental A&E service during the COVID‑19 pandemic in the Greater London area	Emergency service provided by the staff in the Pediatric Dentistry	Patients between 25th March and 29th May 2020 of pediatric dental emergency service	Four-hundred and sixty-four patients	England	2020	A prospective service evaluation	COVID‑19 pandemic	Methods used in a study that evaluated the pediatric dental emergency service during the first lockdown period due to the COVID-19 pandemic. The researchers got approval from the local governance protocol and included all patients who contacted the service by phone or in person from March 25 to May 29, 2020 Replaced the walk-in service with a phone triage system, using a dental emergency form, a medical history form, and a COVID-19 questionnaire. They asked parents to send digital photos of their child's dental problem when needed to help with the assessment. They classified the cases into emergency, urgent, or routine based on their dental and medical needs and arranged face-to-face appointments, referrals, or advice accordingly. Recorded the data of each case using a secure data collection tool in Microsoft Excel, including the patient age, referral source, reason for accessing the service, and triage outcome	During the period of the service evaluation, 464 patients called the emergency service for children's dental problems. Out of these, 192 (41%) had toothache, 121 (26%) had swelling and pain caused by dental issues, and 89 (19%) had injuries to their teeth	Phone calls and digital photos helped to identify urgent dental problems in children, but not being able to see them in person, take X-rays, or use general anesthesia were big challenges
4	Raluca Iurcov/ Pediatric Dental Emergencies during the COVID-19 Pandemic in Romania: A Retrospective Study	The study compared the demographic characteristics of children and teenagers who received emergency dental treatment in Oradea, Romania in 2019, 2020, and 2021, before, during, and after the lockdown	patients treated in the dental emergency department	591 child	Romania	2019–2021	Retrospective Study	COVID-19 Pandemic	The study followed ethical considerations in accordance with the Declaration of Helsinki and obtained parental or legal guardian consent for underage patients. Adult patients provided their own consent. The study was approved by the Ethics Committee. The data was collected retrospectively from medical files of children and adolescent patients registered at the dental emergency department. The department provided free emergency dental treatments. The study analyzed medical files from prelockdown, lockdown, and postlockdown periods. Medical records of patients aged 19 years or older and those lacking relevant information were excluded. Variables such as gender, age group, living environment, location of affected teeth, and type of dental emergency were investigated and recorded in a Microsoft Excel document. Two authors independently verified the medical records to avoid bias. Statistical analysis was conducted using Microsoft Office Excel/Word 2013 and IBM SPSS Statistics 25. Qualitative variables were reported as absolutes or percentages, and Fisher's exact test and Z tests with Bonferroni correction were used for comparisons. Statistical significance was set at *p* < 0.05	The number of children and teenagers who received emergency dental treatment decreased from 257 in 2019 to 136 in 2021. The majority of the patients in each year were 7–12 years old (2019—47.9%; 2020—50.5%; 2021—43.4%), and the lower back teeth were the most commonly affected (2019—53.3%; 2020—53%; 2021—48.5%). The main types of emergencies were inflammation of the pulp (2019—40.5%; 2020—43.9%) and infection of the root tip (2021—42.6%). The data showed that in 2019, younger patients (0–6 years old) had more injuries to their teeth (*p* < 0.001), and in 2019 and 2020, older patients (7–12 years old) had more problems with their gums (*p* < 0.001). In 2020, patients who lived in rural areas had more pulp inflammation (*p* = 0.025), and in 2021, patients who lived in rural areas had more pulp inflammation or root tip infection, while patients who lived in urban areas had more gum problems (*p* = 0.042)	Children who were 7–12 years old had the most dental problems. The most common conditions were inflammation or infection of the pulpitis or acute Apical periodontitis, and they affected the posterior teeth in both the upper and lower jaws the most
5	Giampiero Rossi-Fedele/ Traumatic dental injuries presenting to a pediatric emergency department in a tertiary children's hospital, Adelaide, Australia	The study reviewed the injuries to the teeth that came to the emergency service at the Women's and Children's Hospital in Adelaide (Australia) and identified the features of the patients, accidents, injuries, and treatments	pediatric patients attending the Pediatric Emergency Department (PED) for the management of TDI over 18 months were prospectively reviewed	337 patients	Australia	2020	This prospective study	The patient, accident, injury	The methods used in a retrospective study that analyzed the medical files of children and adolescents who had dental emergencies in three different periods: prelockdown, lockdown, and postlockdown. The study followed ethical principles and obtained consent from the patients or their guardians. The study investigated several variables related to the patients' characteristics, the location of the affected teeth, and the type of dental emergency. The data were organized in Excel and analyzed using SPSS and Fisher's exact test The researchers conducted a retrospective study that looked at the medical records of young patients who needed dental treatments in emergencies in three time periods: before, during, and after the lockdown. The researchers respected ethical guidelines and asked for permission from the patients or their parents. The researchers examined various factors related to the patients' features, the position of the teeth that had problems, and the kind of dental emergency. The data were arranged in Excel and processed using SPSS and Fisher's exact test	The majority of the children who had injuries to their teeth were under 5 years old (56.1%) and boys (63.8%). Most of the accidents happened on weekends (35.6%), at home (48.5%), and were caused by falls (64.4%). A total of 654 teeth were affected, with more baby teeth (58.4%) and upper front teeth (69.9%) involved. The most common type of injury was when the tooth was pushed sideways (27.5%). Most of the children were sent to the Children's Dentistry Department (60.8%). However, many of them did not need any further treatment there and were sent back to their regular dentists (39.2%). The children who did need treatment often had it under general anesthetic (36.9%), and usually had to wait for 3–12 h before getting it (49.1%). The more serious injuries in the adult teeth (when the tooth was knocked out, pulled out, broken at the root, pushed into the bone, or when the bone was broken) also tended to be treated between 3 and 12 h after the accident	Features of the patients, accidents, injuries, and treatments that are similar to what other studies have found in children with dental problems When the adult teeth are injured, they are more likely to be treated within 3 and 12 h without being admitted to the hospital, while when the baby teeth are injured, they are more likely to be treated between 12 and 24 h under general anesthesia
6	Amir Elalouf/ Pediatric Dental Emergency Visits and Treatment during Lockdown in the COVID-19 Pandemic: A Retrospective Study	The study examined the reasons for emergency visits of children to dental clinics and the treatments given during the lockdown	emergency pediatric patient Visits in dental clinics and their treatments before, during, and after the lockdown periods	to 30 April 2020), and after lockdown	Israel	2019 and 2020	A Retrospective Study	COVID-19 Pandemic	Methods of a study that looked at the dental emergencies of young patients in Israel before, during, and after the lockdown. The study used data from Maccabi-Dent, a large dental service provider, and followed ethical and safety protocols. The study examined the patients' characteristics, the reasons for their visits, and the treatments they received. The study used Excel and SPSS to analyze the data and compare the different periods	More children came to the emergency service with pain (*n* = 281, 32.6%) than with injuries (*n* = 18, 24.7%) during the lockdown, as well as with infection (*n* = 31, 28.4%) and ongoing treatment (*n* = 7, 20.6%). These numbers were higher than before and after the lockdown periods. There was a significant difference (*p* < 0.001) in the medication and dental procedures given to the children during the lockdown compared to before and after the lockdown. Pulling out teeth (*n* = 81, 41.5%), filling cavities (*n* = 84, 50.6%), applying fluoride (*n* = 13, 92.9%), and treating the pulp (*n* = 92, 42.6%) were the main treatments done for the children during the lockdown	Professional dentists quickly adjusted to treat children who were not vaccinated and learned how to better prepare and meet the future challenges of such situations
7	Marilisa Toma/ Dental Emergencies in an Italian Pediatric Hospital during the COVID-19 Pandemic	The study analyzed the number and cause of visits at the emergency room (ER) of Ospedale dei Bambini “Vittore Buzzi”, the main children's hospital in Milano, Italy, between 2019 and 2020	ER register of Ospedale dei Bambini “Vittore Buzzi”, ASST Fatebenefratelli-Sacco, Milano, Italy, during the period between 1 January 2019 and 31 December 2020	children In 2019, 25,435 16,750 in 2020	Italy	between 2019 and 2020	retrospectively analyzed data	COVID-19 Pandemic	Methods of a study that looked at the oral health emergencies of children who visited the ER of a hospital in Italy before and during the COVID-19 pandemic. The study used data from the ER register, which recorded information about the date, time, gender, reason, triage code, and final diagnosis of each visit. Searched the files using keywords related to oral health problems and categorized them into four groups: stomatitis/aphthous stomatitis, abscesses/infections, painful caries, and trauma. The study only analyzed the data of the non-COVID-19 cases and compared the numbers of visits between 2019 and 2020	The same percentage of children (10%) had to stay in the hospital after visiting the ER in both years. there were fewer cases of infectious diseases, except for COVID-19, in 2020 than in 2019, while the number of injuries decreased slightly but the proportion increased. fewer children came to the ER for oral problems in 2020 than in 2019. The most common oral problem was inflammation of the mouth, followed by injuries to the teeth, which were less frequent and less common in 2020 than in 2019. Approximately 15% of the oral problems in both years were caused by infections or decayed teeth that caused pain	There is a need to improve the local services that can prevent and treat oral health problems, including dental emergencies

## Discussion

All of articles focus on the oral and dental health of children in different situations, such as disasters, pandemics, accidents, or emergencies, use quantitative data, such as surveys, records, or statistics, to describe or analyze the oral and dental problems and treatments of children, report on the prevalence, incidence, patterns, or causes of oral and dental problems and treatments of children, such as pain, infection, inflammation, injury, or extraction, suggest some ways to improve the oral and dental health and care of children, such as increasing the awareness and education of dentists and parents, developing and implementing guidelines and protocols, improving the coordination and collaboration among dental services and other health professionals, and conducting more research and evaluation.

They focus on different aspects of oral and dental health and care of children, such as estimating the age of individuals, coping with the COVID-19 pandemic, identifying urgent dental problems, analyzing emergency dental treatment, reviewing dental injury features, reporting on dental adjustment during the lockdown, or comparing oral problems in the ER, use different sources, samples, or periods of data, such as dental development methods, pedodontist surveys, phone and digital photo service, hospital records, literature review, professional dentist reports, or ER data, report different results or findings, such as the limitations of the Cameriere method, the difficulties of wearing PPE, the challenges of not seeing the patients in person, the decrease of patients from 2019 to 2021, the similarity of injury features to other studies, the difference of medication and procedures during the lockdown, or the decrease of oral problems in 2020, suggest different implications or recommendations, such as using both the dental and skeletal methods together, working longer hours or charging more for services, using video calls or providing more training and support, educating and communicating with children and parents, applying innovative and regenerative approaches, or improving the local services.

Out of the 7 articles included in the study, 5 were related to the conditions of the coronavirus pandemic, and two other articles were related to the identification of victims and the prevalence of child referrals to specialized emergency rooms due to head and neck accidents. These articles show how dentistry for children plays a vital role in various types of disasters by providing emergency dental services, participating in the identification of victims through dental evidence, or supporting the public health and well-being of children in disaster situations. They also show how disasters pose challenges and risks to oral health and child care, as well as to dental practice and education. They highlight the need for more research and evidence-based interventions to improve children's oral health in disaster settings [31, 32]. The COVID-19 pandemic has created unprecedented challenges and risks for the dental profession, especially pediatric dentistry. The purpose of this text is to review articles on the impact of COVID-19 on pediatric dental emergencies, services, and knowledge in different countries and environments. Pediatric dental emergencies are conditions that require immediate attention to relieve pain, infection, or trauma. During the COVID-19 pandemic, many dental clinics were closed or restricted to providing only emergency care, according to WHO guidelines [31, 33]. However, the definition and management of pediatric dental emergencies vary across countries and regions, depending on the availability of resources, infection control measures, and referral systems [34–36].

Several studies have reported the characteristics and outcomes of pediatric dental emergencies during the COVID-19 pandemic in countries as diverse as Italy, Israel, Romania, and the United Kingdom. These studies have shown that the number and proportion of pediatric dental emergencies during quarantine periods have decreased compared to prepandemic periods. The most common causes of pediatric dental emergencies were trauma, caries, and infection, mainly affecting the anterior teeth of both teeth. The most common treatments offered were extractions, pulpotomies, and fillings. These studies also revealed some of the challenges and barriers to providing pediatric emergency dental care during the pandemic, such as a lack of personal protective equipment (PPE), limited access to general anesthesia, delayed referrals, and parental fear of infection [26–28, 30].

In addition to providing emergency care, pediatric dentists also play a role in preventing and controlling the spread of COVID-19 among children and their families. A study conducted in India assessed the knowledge and understanding of COVID-19 among pediatricians (pediatric dental specialists) using an online survey. The results showed that most pediatricians had sufficient knowledge and a positive understanding of COVID-19 and its prevention measures. However, some gaps and misconceptions were also identified, such as the mode of transmission, incubation period, and symptoms of COVID-19. The study suggested that pediatricians should regularly update their knowledge and skills and follow evidence-based guidelines and recommendations for performing dental practice during the pandemic [29, 37, 38].

The role of dentistry for children during COVID-19 is very important to maintain their oral health and wellness as well as prevent further complications. Pediatric dentists should be aware of the current situation and the challenges caused by the pandemic and adapt their practice accordingly. They should also educate and communicate with children and their parents about the importance of oral hygiene, a healthy diet, and preventive measures to reduce the risk of contracting a COVID-19 infection. In addition, they must collaborate with other health professionals and authorities to ensure the safety and quality of children's dental care during and after the pandemic [24, 39, 40].

Dentistry for children is important not only for maintaining their oral health and wellness but also for providing valuable information for disaster victim identification (DVI). DVI is the process of identifying human remains after massive disasters such as earthquakes, tsunamis, or terrorist attacks. Pediatric dentistry can help DVI in two ways: by recording and documenting traumatic injuries to the teeth and cervical vertebrae. The stages of dental maturity and cervical vertebrae are indicators of biological age that can be used to estimate the age of unknown victims. Dental maturity refers to the growth and eruption of teeth, while cervical vertebral maturity refers to the growth and shape of the cervical vertebrae. Dental maturity and cervical vertebrae can be assessed with radiographs, such as panoramic or lateral cephalometric images. A study by Timmins et al. evaluated the usefulness of stages of dental maturation and cervical vertebrae in New Zealand children for DVI. This study showed that dental maturity is more accurate in age estimation than cervical vertebral maturity, and both methods are more reliable in younger children than in older children. The study also suggested that the stages of dental and cervical maturation should be standardized and included in children's dental records. Traumatic dental injuries (TDIs) are common in children and can affect the appearance and function of their teeth. TDI can also provide distinctive features that can help identify victims after disasters. Depending on the extent and location of the injury, TDI can be classified into several types, including crown fracture, luxation, or alveolar fracture. A study by Ng et al. investigated the characteristics and outcomes of TDI presentations to the pediatric emergency department at a tertiary children's hospital in Adelaide, Australia. This study showed that TDI occurs more often in boys than in girls, and most of the lesions involve maxillary incisors. This study also showed that most of the injuries were caused by falls or collisions, and most of the treatments were extraction, filling, or pulpotomy. This study recommended that TDI should be documented in detail and regularly followed up by dentists. Consequently, pediatric dentistry plays a vital role in DVI by providing information on dental and cervical maturation stages and TDI. This information can help narrow the search for missing people and match them to their dental records. Therefore, pediatric dentists should be aware of the importance of recording and updating this information in their office [24, 25, 41, 42].

One limitation of the study was using only English articles.

### Suggestions for the future

There seems to be a lack of a comprehensive protocol that can define the role of dentistry in accidents and disasters for vulnerable people, such as children. This issue requires more detailed and comprehensive investigations, which can include a part of a comprehensive program for vulnerable people in accidents and disasters that can profitably benefit from the field of dentistry. The damage caused by accidents and disasters can include different degrees, and often, except for death, with quick and timely action, the damage can be reduced to a larger volume, which requires continuous monitoring of global information and data in an up-to-date manner. Here is a possible paraphrase of the paragraph:

- The global situation for Disasters in pediatric dentistry is not easy or clear to solve, as different regions and countries may have different problems and opportunities.

Some possible ideas for improving the role and impact of dentistry in helping children affected by disasters are:

- Making dentists, other health professionals, and authorities more aware and educated about the importance of oral and dental health for children in disaster situations, and the possible risks and complications of dentofacial injuries.
- Creating and applying guidelines, protocols, and standards for giving emergency and comprehensive dental care for children in disaster situations, including infection control, pain management, trauma management, and restoration of function and aesthetics.
- Making the coordination and collaboration among dentists, other health professionals, and authorities better in disaster preparedness, response, and recovery, and making sure the availability and accessibility of dental personnel, facilities, and equipment.
- Identifying and referring children with possible chronic difficulties, such as developmental disabilities, dental phobia, child maltreatment, or mental health problems that may harm their oral and dental health or need special dental care.
- Teaching and communicating with children and their parents about the importance of oral hygiene and healthy diet, and giving them the necessary information, support, and resources to prevent or reduce dental problems.
- Doing research and collecting data on the epidemiology, patterns, and outcomes of dentofacial injuries and other oral and dental problems in children in disaster situations, and evaluating the effectiveness and quality of dental care given.

## Conclusion

Dentistry has a unique opportunity to help in various aspects of diagnosis and treatment relief, besides reducing the number of deaths and injuries. This special feature of dentistry requires more attention to planning, protocols, and actions before the disaster occurs. Dentistry can also help identify the victims.

## Data Availability

The datasets used during the current study available from the corresponding author on reasonable request.
